# Endoscopic features of lymphoid follicles in the colonic mucosa using the image enhanced endoscopy and its association with colorectal adenoma

**DOI:** 10.1371/journal.pone.0286300

**Published:** 2023-05-30

**Authors:** Tomomitsu Tahara, Kazuya Takahama, Sayumi Tahara, Noriyuki Horiguchi, Kohei Funasaka, Yoshihito Nakagawa, Tomoyuki Shibata, Tetsuya Tsukamoto, Hiro-o Ieda, Toshiro Fukui, Makoto Naganuma, Naoki Ohmiya

**Affiliations:** 1 Third department of Gastroenterology, Kansai Medical University, Hirakata, Japan; 2 Department of Gastroenterology I, Fujita Health University School of Medicine, Toyoake, Japan; 3 Internal Medicine, Takahama Clinic, Nishio, Japan; 4 Endoscopic Center, Ieda Hospital, Toyota, Japan; 5 Department of Diagnostic Pathology, School of Medicine, Fujita Health University, Toyoake, Japan; 6 Department of Advanced Endoscopy, Fujita Health University, Toyoake, Aichi, Japan; The University of Hong Kong Li Ka Shing Faculty of Medicine, HONG KONG

## Abstract

**Background/Aim:**

Lymphoid follicles hyperplasia (LH) is sometimes observed in the normal colon as small, round, yellowish-white nodules. LH is associated with food hypersensitivity and bowel symptoms and histologically characterized as intense infiltration of lymphocytes or plasmacytes. It is suggested that LH represents inflammatory immune response in the colonic mucosa. We investigated the presence of LH in the normal colonic mucosa and its association with incidence of colorectal lesions including colorectal cancer, adenoma and hyperplastic polyp.

**Patients/Methods:**

605 participants undergoing colonoscopy for various indications were enrolled. Presence of LH in the proximal colon (appendix, cecum and the ascending colon) was observed using the blue laser imaging (BLI) endoscopy, a new generation image enhanced endoscopy (IEE) system. LH was defined as well demarcated white nodules. Elevated LH with erythema was distinguished as LH severe. Association between presence of LH and occurrence of colorectal lesions was investigated.

**Results:**

Prevalence of all colorectal lesions and adenoma were significantly lower in LH severe group compared to the LH negative group (*P* = 0.0008, 0.0009, respectively). Mean number of all colorectal lesions and adenoma were also lower in LH severe group compared to the LH negative group (*P* = 0.005, 0.003 respectively). The logistic regression with adjustment for gender and age demonstrated that presence of LH severe held significantly lower risk of all colorectal lesions (OR = 0.48, 95%CI = 0.27–0.86) and adenoma (OR = 0.47, 95%CI = 0.26–0.86).

**Conclusion:**

LH in the colonic mucosa visualized by IEE is useful endoscopic finding to predict risk of colorectal adenoma.

## Introduction

Colorectal cancer (CRC) is the third most common cancer in men and the second in women worldwide [1]. Colonoscopy is considered as the gold standard for diagnosis and surveillance of colorectal neoplasms. Emerging data support the evidence that colonoscopy reduces the incidence and mortality of CRC by detection and removal of adenomatous polyps [2]. However, considering the cost and invasiveness of colonoscopy, optimizing intervals for surveillance colonoscopy, reflecting individual risk would be ideal. It has been reported that lifestyle factors, such as alcohol consumption, obesity, and physical inactivity is associated with the risk of developing CRC [3], while host genetic factor and microbiota have been also associated with risk of CRC [4, 5]. It is therefore possible that complex interaction between environmental, host genetic and bacterial factors define individual inflammatory immune responses in the colonic mucosa and influence the risk of CRC.

In the colorectum, small, round, yellowish-white nodules, which is presumably an endoscopic feature of lymphoid follicles hyperplasia (LH), are sometimes observed. LHs in the small and large intestine was associated with food hypersensitivity and bowel symptoms [6]. LH is histologically characterized as intense infiltration of lymphocytes or plasmacytes [7]. LHs may therefore reflect individual inflammatory immune responses in the colonic mucosa. We have previously reported that LHs are observed more clearly by using blue laser imaging (BLI) endoscopy, a new generation image-enhanced endoscopy (IEE) system and correlated well with chronic bowel symptoms in healthy subjects [7]. Since colorectal tumorigenesis involves complex inflammatory immune responses that are still not completely understood, we investigated the potential association between occurrence of colorectal lesions including CRC, adenoma and hyperplastic polyp and presence of LHs visualized by the BLI endoscopy.

## Methods

### Ethics statement

This study was approved by the Human Research Ethics Committee of the Fujita Health University School of Medicine (ID: HM16-250). Each participant provided written informed consent for their clinical and laboratory data to be used and published for research purposes. The study was conducted according to the principles expressed in the Declaration of Helsinki.

### Study population

Study participants were prospectively enrolled from patients of the endoscopy Center of Fujita Health University and Ieda Hospital from December 2014 to December 2018. Six hundred and eight participants were initially invited, and all agreed to participate. All participants underwent total colonoscopy for various indications, including fecal immunological test positive, yearly follow up examination screening, concern about colonic diseases. Excluded from the study were patients who had severe systemic disease, inflammatory bowel diseases, Celiac disease, acute infection including acute enteritis and those who had had a history of abdominal surgery. After total colonoscopy, two and one patients were excluded for suspicious of ulcerative colitis and Crohn’s Disease. Finally, six hundred and five participants were included for the study. Fujita Health University School of Medicine approved the protocol, and written informed consent was obtained from all participants.

### Endoscopic procedure and definition of LH

**Fig 1:**
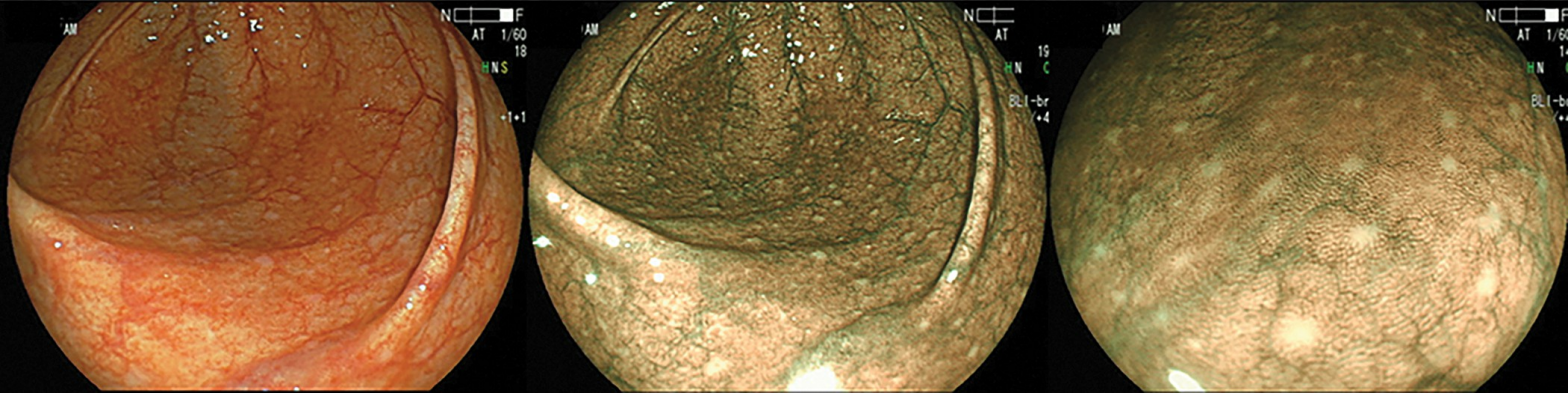
Lymphoid follicles hyperplasia (LH), observed by the conventional white light (left) and BLI-bright mode without magnification (center) and with magnification (right). LHs are observed as well demarcated white nodules.

All participants took 1.5 L polyethylene glycol solution (MOVIPREP; EA Pharma Co., Tokyo, Japan) on the morning before the examination to clean their entire colon. The video endoscope used in this study was an EC-L590ZW (FUJIFILM Corporation, Tokyo, Japan). After inserting colonoscope into the cecum, presence of LH in the proximal colon (appendix, cecum and the ascending colon) was observed with the BLI light source being turned on. The BLI has a semiconductor laser as a light source. Principally, the system includes two types of lasers with wavelengths of 410 and 450-nm. The 450-nm laser irradiates phosphor to produce illumination light similar to that obtained with a xenon lamp. The combination of strong 410-nm laser light, weak 450-nm laser light and fluorescent light enables blue laser imaging (BLI) via narrow-band light observation. Moreover, the BLI has bright mode, which achieves a brighter image than usual BLI mode to maintain the enhanced contrast of surface vessels from a far field of view. It has been reported that LHs in the colonic mucosa, predominantly observed in the appendix (cecum) and the ascending colon, can be visualized more clearly by using BLI-bright mode compared to conventional white light colonoscopy [7] (Fig 1). We therefore used the BLI-bright mode to evaluate the presence of LH in all cases.

**Fig 2:**
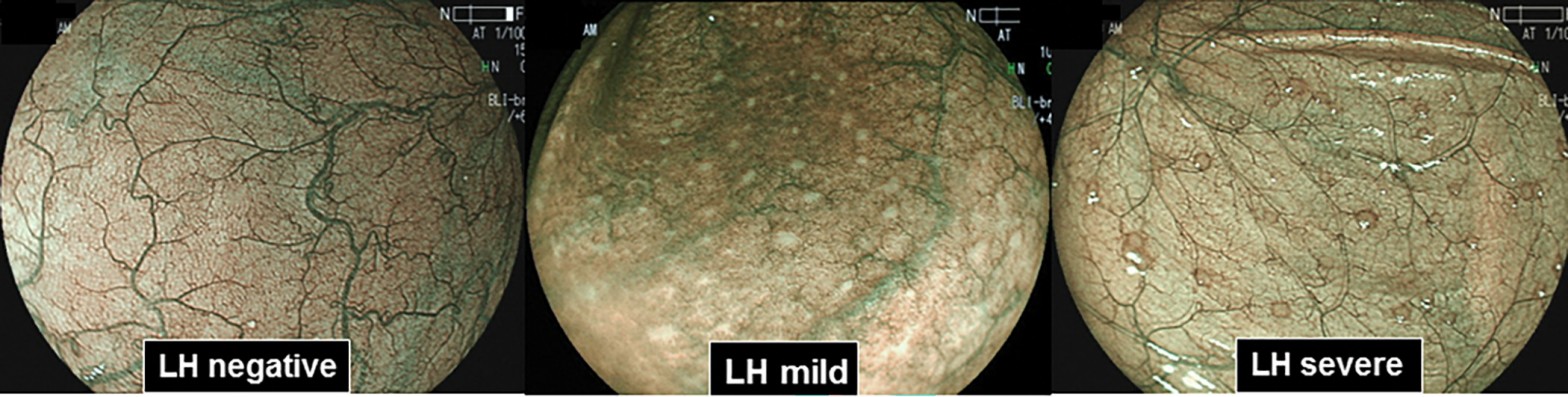
Representative endoscopic BLI findings of LH negative (left), mild (center) and severe (right). Elevated LH with erythema was distinguished as LH severe (right). All other LHs were considered as LH mild (center).

According to the previous report [7], the presence of LH was defined as well demarcated white nodules. Elevated LH with erythematous change was distinguished as LH severe (Fig 2). Although we mainly used BLI light with non-magnification view, presence of LH was also confirmed using the magnification view. After the evaluation of LH, entire colorectum was also evaluated for the presence of colorectal lesions such as polyps and cancer. During the examination, at least 40 photographs were taken from the entire colorectum, even if there was no obvious lesion. If the obvious lesion such as polyps and cancer were seen, the location and size of all detected lesions were recorded. The lesion size was estimated by using open endoscopic biopsy forceps and/or a snare. For the evaluation of polyps and cancer, we mainly used conventional white light but if necessary, BLI light was also used. All lesions that the endoscopist diagnosed as adenomas or hyperplastic polyps >10 mm was removed by hot biopsy, hot snare polypectomy or EMR, while biopsies were taken from all lesions that the endoscopist diagnosed as hyperplastic polyps <10 mm. Biopsies were also taken from lesions that the endoscopist diagnosed as invasive cancer. Endoscopic examinations were performed by a single expert endoscopist (T.T). Using the endoscopic photographs, presence of LH in each case was judged by the consensus manner among the three expert endoscopists (T.T, K.T and M.O). All resected specimens were fixed in 10% buffered formalin solution and examined histologically by using hematoxylin and eosin staining. Experienced pathologists who were blinded to the endoscopic diagnosis performed the histopathologic diagnosis.

### Statistical analysis

Categorical values among different groups were compared using the Chi Square Statistics.

Continuous values among two and three different groups were compared using the Student’s t-test. The odds ratios (OR) and 95% confidence intervals (CI) were also calculated by the logistic regression with adjustment for age and gender. A P value of less than 0.05 was considered statistically significant.

## Results

### LH observed by BLI colonoscopy and its association with clinic-pathological features

Clinic-pathological features of Six hundred and five subjects are shown in the Table 1.

**Table 1:** Clinicopathological characteristics of patients.

Variables (n)	
*Gender*: male/female	376/229
*Age*: median (range)	61 (22–84)
*Reason for colonoscopy*	
Fecal blood test positive	228
Follow up examination	189
Concern about bowel disease	108
Others	80

For all subjects, presence of LH in the proximal colon (appendix, cecum and the ascending colon) was evaluated using BLI bright mode (Fig 1). In overall subjects, LH was observed in 238 subjects (39.3%). LHs were also divided into two groups according to the presence of clear elevation and erythema: LH severe and LH mild (Fig 2). 115 (19.0%) were considered as LH severe. We then investigated the association between presence of LH with gender, age and reasons for colonoscopy. Presence of LH, especially the LH severe was significantly associated with female gender and younger age. On the other hand, no association was found between the presence of LH and reason for colonoscopy (S1 Table).

### Association between presence of LH and occurrence of colorectal lesions

We investigated the association between presence of LH and occurrence of colorectal lesions. Presence of colorectal lesions were investigated throughout the colorectum for all hundred subjects. In total, 316 colorectal lesions were found in 162 patients (26.8%) and all remaining did not have any colorectal lesions. Summary of 316 lesions including histology, location and size are shown in the Table 2. Among 268 adenoma lesions, histological assessment revealed that two were adenoma with high-grade dysplasia and remaining were adenoma with low-grade dysplasia according to the World Health Organization criteria [8].

**Table 2:** Summary of 316 colorectal lesions found in 162 patients.

Variables	
*Histology*	
Invasive cancer	6
Adenoma	268
Hyperplastic polyp	42
*Location*	
Cecum and ascending colon	68
Transvers colon	50
Descending colon	23
Sigmoid colon	140
Rectum	24
*Size* mean (range)	5.03 (2–40) mm

Location and size were not determined in 11 and 26 lesions, respectively.

Prevalence of all colorectal lesions including invasive cancer, adenoma and hyperplastic polyp was 31.1% (114/367), 25.2% (31/123) and 14.8% (17/115) for LH negative, mild and severe subjects, respectively. Prevalence of all colorectal lesions was significantly lower in LH severe group compared to the LH negative group (*P* = 0.0008) (Table 3). We also investigated the association between presence of LH and occurrence of invasive cancer, adenoma and hyperplastic polyp. Prevalence of adenoma was significantly lower in LH severe group compared to the LH negative group (*P* = 0.0009), while such association was not found for invasive cancer and hyperplastic polyp (Table 3). We also investigated the association between presence of LH and mean number of colorectal lesions and found that mean number of all colorectal lesions and adenoma were significantly lower in LH severe group compared to the LH negative group (*P* = 0.005, 0.003 respectively) (Table 4).

**Table 3:** Association between presence of LH and occurrence of colorectal lesions.

Variables (n)	All colorectal lesions*	Invasive cancer	Adenoma**	Hyperplastic polyp
	n (%)	n (%)	n (%)	n (%)
LH negative (n = 367)	114 (31.1%)	4 (1.1%)	106 (28.9%)	12 (3.3%)
LH mild (n = 123)	31 (25.2%)	2 (1.6%)	26 (21.1%)	8 (6.5%)
LH severe (n = 115)	17 (14.8%)	0 (0%)	15 (13.0%)	3 (2.6%)

* LH negative *vs*. LH severe, *P* = 0.0008

** LH negative *vs*. LH severe, *P* = 0.0009.

Statistical analysis was performed by the chi-square test.

**Table 4:** Association between presence of LH and mean number of colorectal lesions.

Variables (n)	All lesions*	Invasive cancer	Adenoma**	Hyperplastic polyp
	Mean +/- SE	Mean +/- SE	Mean +/- SE	Mean +/- SE
LH negative (n = 367)	0.61+/-0.06	0.11+/-0.01	0.54+/-0.06	0.05+/-0.02
LH mild (n = 123)	0.49+/-0.10	0.02+/-0.01	0.34+/-0.07	0.13+/-0.05
LH severe (n = 115)	0.29+/-0.08	0.0+/-0.0	0.24+/-0.07	0.05+/-0.03

* LH negative *vs*. LH severe, *P* = 0.005

** LH negative *vs*. LH severe, *P* = 0.003.

Statistical analysis was performed by the Student’s t-test.

Since presence of LH was significantly associated with female gender and younger age (S1 Table). Association between presence of LH and occurrence of colorectal lesions was evaluated by the logistic regression with adjustment for gender and age. The result demonstrated that presence of LH severe held significantly lower risk of all colorectal lesions (adjusted OR = 0.48, 95%CI = 0.27–0.86, *P* = 0.01) and adenoma (adjusted OR = 0.47, 95%CI = 0.26–0.86, *P* = 0.01) (Table 5). We also investigated whether the presence of LH would be associated with size of adenoma in patients who had adenoma lesions. However, no association was found between presence of LH and size of adenomas (S2 Table).

**Table 5:** Multivariate analysis assessing factors associated with occurrence of all colorectal lesions and adenoma.

Variables	All colorectal lesions	Adenoma
	OR (95%CI) *P*	OR (95%CI) *P*
Gender (male)	1.51 (1.02–2.23) 0.04	1.72 (1.14–2.60) 0.01
Age	1.02 (1.01–1.04) 0.003	1.03 (1.01–1.04) 0.003
LH mild	0.81 (0.51–1.29) 0.38	0.71 (0.44–1.17) 0.38
LH severe	0.48 (0.27–0.86) 0.01	0.47 (0.26–0.86) 0.01

## Discussion

LHs in the small and large intestine was associated with food hypersensitivity and bowel symptoms [6]. It has been reported that LHs are histologically characterized as intense infiltration of lymphocytes or plasmacytes [7], suggesting that LHs represents inflammatory immune response in the colonic mucosa. The role of inflammatory immune response in the development of colorectal neoplasms has been suggested from positive association between CRC and specific types of microbiota, most notably the *Fusobacterium* species [5, 9, 10], which have invasive [11], adherent [12], and pro-inflammatory [13] features. In the present study, we investigated the clinical relevance of LHs in relation to the occurrence of colorectal lesions. We showed that presence of LHs in the proximal colon (appendix, cecum and the ascending colon) was correlated with occurrence of colorectal lesions. We showed that LH severe, characterized as elevated LH with erythema was negatively correlated with occurrence and number of colorectal lesions especially adenomas. So far, colonic mucosal inflammatory immune response has been recognized as an important mechanism in colorectal neoplasms, but direct link with endoscopically visualized LHs has not been clearly reported, probably due to the lack of sensitivity to detect LHs by the conventional white light colonoscopy. It has been reported that LHs in the gastrointestinal tract can be clearly visualized using IEE systems such as Narrow Band Imaging (NBI) and BLI endoscopy [7, 14]. We used BLI with bright mode, which achieves a brighter image than usual mode to maintain the enhanced contrast of surface vessels from a far field of view [15]. Our result provided the evidence that presence of LHs using the BLI colonoscopy is useful endoscopic appearance to predict occurrence of colorectal neoplasms such as adenomas. Presence of LH has been correlated with food hypersensitivity and bowel symptoms in the younger generations [6]. Positive association between presence of LH and younger age was also observed in our study. It is possible that LHs decrease by age, while patients who remain LHs in the adulthood may have decreased risk of colorectal adenomas. On the other hand, it would be usual that the younger patients don’t typically undergo screening colonoscopy unless they have symptoms. Although the association between LH and colorectal lesions was confirmed by the logistic regression with adjustment for age, it needs to be investigated the prevalence of LHs in the larger cohort of younger patients. We also showed that LH was more frequently observed in female subjects. Since overall incidence and mortality rates for CRC are substantially lower for females [1]. We speculate that higher frequency of LHs in the female subjects may be associated with decreased risk of CRC in the female gender, reflecting difference of inflammatory immune response On the other hand, it should be also noted that the lifestyle differences in different gender such as alcohol consumption and diet may influence this issue.

Regarding the subtypes of LHs, we have shown that LH severe, but not mild was significantly associated with decreased risk of colorectal lesions especially adenomas. This indicates the importance of discriminating the appearances or locations of LHs to predict risk of colorectal lesions. Our data suggest that LH severe may reflect stronger inflammatory immune response that may be more closely associated with decreased risk of colorectal lesions. Mechanistic study will be also needed to clarify how the LH reflects inflammatory immune response that prevent colorectal neoplasms. This is a preliminary data reporting the potential association between LH and colorectal neoplasms, but our findings raise the possibility of important role of this endoscopic finding in the colorectal neoplasms, opening the avenue of research on IEE techniques in this field. We believe that our result provide salient findings despite it is a preliminary investigation.

## Supporting information

S1 Table Clinicopathological characteristics of LH negative, mild and severe cases. (DOCX)

S2 Table Presence of LH and size of adenoma in cases who had adenoma lesion. (DOCX)
