# Supplementary material for: Endoscopic features of lymphoid follicles in the colonic mucosa using the image enhanced endoscopy and its association with colorectal adenoma
Source: PLoS One. 2023 May 30;18(5):e0286300. doi: 10.1371/journal.pone.0286300 (PMC10228764; doi:10.1371/journal.pone.0286300)
Supplement: S1 Table — (DOCX) [file pone.0286300.s001.docx]

**Supporting information**

**S1 table** Clinicopathological characteristics of　LH negative, mild and severe cases

| Variables (n) | Gender * | Age** | Reason for colonoscopy |
| --- | --- | --- | --- |
|  | male/ female | median (range) | Fecal blood test positive/follow up/concern about bowel disease/ others |
| LH negative (n=367) | 241/126 | 64 (25-83) | 132/128/62/45 |
| LH mild (n=123) | 74/49 | 60 (24-83) | 54/30/20/19 |
| LH severe (n=115) | 61/54 | 54 (22-84) | 42/31/26/16 |
| * LH negative *vs.* LH severe, *P*=0.01;  ** LH negative *vs.* LH mild, *P*=0.02; LH negative *vs.* LH severe, *P*<0.0001; LH mild *vs.* LH severe, *P*=0.001;  * Statistical analysis was performed by the Student's t-test. ** Statistical analysis was performed by the chi-square test. | | | |
