# Supplementary material for: Endoscopic features of lymphoid follicles in the colonic mucosa using the image enhanced endoscopy and its association with colorectal adenoma
Source: PLoS One. 2023 May 30;18(5):e0286300. doi: 10.1371/journal.pone.0286300 (PMC10228764; doi:10.1371/journal.pone.0286300)
Supplement: S2 Table — (DOCX) [file pone.0286300.s002.docx]

**Supporting information**

**S2 table**  Presence of LH and size of

adenoma in cases who had adenoma lesion

| Variables (n) | Size of adenoma |
| --- | --- |
|  | Mean +/- SE mm |
| LH negative (n=102) | 5.56+/-0.41 |
| LH mild (n=26) | 5.70+/-0.54 |
| LH severe (n=14) | 5.93+/-0.98 |
| Size was not available in 2 and 3 cases with | |
| LH negative and severe cases, respectively. | |
